# Supplementary material for: Zero is not absence: censoring-based differential abundance analysis for microbiome data
Source: Bioinformatics. 2024 Feb 8;40(2):btae071. doi: 10.1093/bioinformatics/btae071 (PMC10885211; doi:10.1093/bioinformatics/btae071)
Supplement: btae071_Supplementary_Data [file btae071_supplementary_data.pdf]

S1 Role of negative log transformation

Following Section 2 in Methods, the negative log transformation is used to map left-censored $(0, 1)$ data to right-censored $(0, \infty)$ data. In response to a reviewer's inquiry about whether this transformation is unique or optimal, here, we offer additional clarification. Although various transformations are theoretically possible, the negative log transformation was selected for its effectiveness in addressing the skewness characteristic of relative abundance data, thereby making it more amenable to standard parametric models. This transformation is well-studied and commonly used in statistical analysis due to its capability to effectively normalize data distributions. Nonetheless, defining an "optimal" transformation can be challenging and is highly dependent on the specific context. We acknowledge that exploring alternative transformations presents a valuable avenue for future research, aiming to further enhance data analysis in this domain.

S2 Sensitivity to choice of d

Building on the previous section's approach, we also utilized Simulation 1, with initial library size generated from Unif$(25000, 40000)$ for both groups. However, we varied the value of d used in practice from $d = 0.5$ to $d = 1, 2, 3, 4$, and 5. The choices of $d = 0.5$ and $d = 1$ are particularly noteworthy, as one reviewer highlighted $d = 0.5$ as an optimal choice from a measurement error perspective. In Figure S1, we observe that the choice of d has a relatively minimal effect on type I error (Panel A). However, it significantly influences power (Panel B), with larger values of d leading to reduced power. This reduction in power may stem from the introduction of excessive noise when "imputing" non-zero entries with larger entries, potentially obscuring the distinction between non-null and null taxa. Nevertheless, results for $d = 0.5$ and $d = 1$ are very similar,

indicating no substantial practical difference between these values. From an interpretative standpoint, we recommend using $d = 1$, as it represents a more intuitive censoring cutoff for read counts, whereas $d = 0.5$ equivalent to half a read−lacks interpretability.

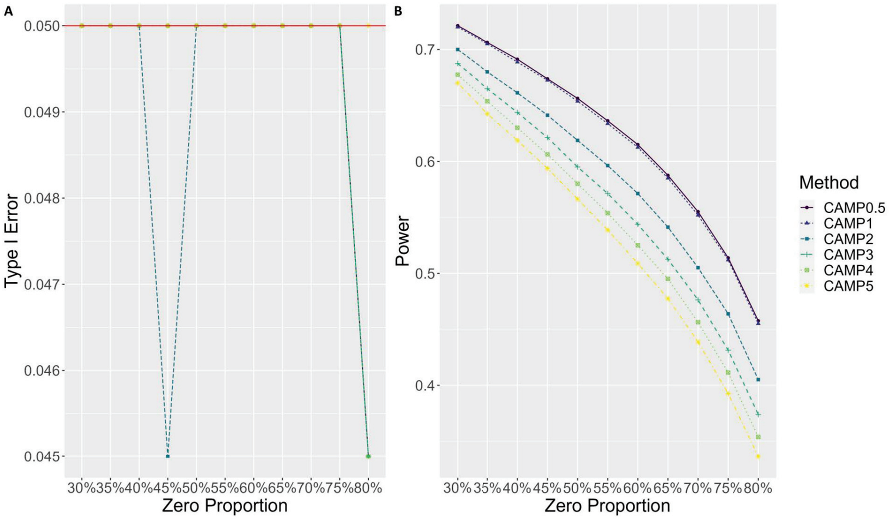

Fig. S1 Median type I error and power for CAMP across 1000 replicates in simulation 1: (A) Type I error; (B) Power.

S3 Proportional hazards assumption

Both the log-rank test and Cox proportional hazards model (the case with covariates) are most powerful under the proportional hazards assumption. Following one reviewer's suggestion, we checked this assumption in the context of negative log-transformed relative abundance data. We used the same preprocessed human gut microbiome data from our real data analysis. For each taxa from the 3 pairwise comparison between countries, we tested the proportional hazards assumption using the Schonenfeld residuals. We applied the Benjamini-Hochberg procedure to the p-values of test and the proportion of

tests rejected at 5% level is presented in Table S1. As shown in Table S1, we failed to reject approximately 90% or more of the tests empirically, suggesting that there are minimal evidence against the proportional hazards assumption and potentially works fine as a working assumption. We hypothesize that there is a biological relevance as to why this assumption may hold: microbial communities can reach a state of relative equilibrium or balance such that the ratio of abundance or presence of different taxa remain relatively constant over abundance level.

Table S1 Proportion of tests that rejected the proportional hazards assumption after correcting for multiple comparison at 5% level in three pairwise comparisons (MA vs US; MA vs VE and US vs VE).

% rejected (MA vs US)	% rejected (MA vs VE)	% rejected (US vs VE)
10.4%	3.7%	11.6%

S4 Performance metrics

We used type I error, power and false discover rate (FDR)

$$\text{Type I error} = \frac{\text{FP}}{\text{TN} + \text{FP}}$$
$$\text{Power} = \frac{\text{TP}}{\text{TP} + \text{FN}}$$
$$\text{FDR} = \frac{\text{FP}}{\text{FP} + \text{TP}}$$

to compare the performance of different DAA methods in simulations 1 to 3, where the number of true positive (TP) is the number of non-null taxa detected at the corresponding p-value threshold. Similarly, the number of true negatives (TN) is the number of null taxa **not** detected at the p-value threshold. Then, the number of false positives (FP) and the number of false negatives (FN)

simply given by the number of non-null taxa minus TP, and the number of null taxa minus TN, respectively. For type I error, we used a 5% level; and we applied Benjamini Hochberg procedure to p-values at 5% level for power and FDR. Notice that FDR is not available for simulation 3 since the null and non-null taxa were generated separately.

S5 Real data analysis results

To complement our main simulations, which were based on data generated under assumed underlying models, we conducted a simulation using the same human gut microbiome data from the real data analysis. More information about this dataset can be found in Section 4 of Methods. After data preprocessing, we performed pairwise DAA by comparing the data from two countries at a time and repeating this process for all $\binom{3}{2} = 3$ country pairs. To assess the type I error rate, we permuted the country labels for each pair.

In this simulation (Figure S2), CAMP, LDM, and MetagenomeSeq were the only methods that consistently controlled the median type I error rate. MetagenomeSeq, however, was slightly more conservative than the other two. The remaining methods—corncob, DESeq2, and ZINQ—exhibited inflated median type I errors, specifically 20%, 12%, and 8%, respectively, across three pairwise comparisons. Once again, this showcases the advantages of employing a non-parametric approach in DAA.

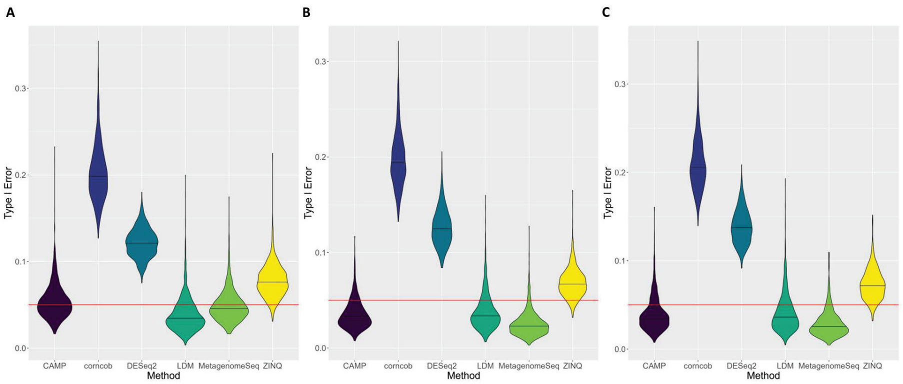

Fig. S2 Type I error for 6 methods across 1000 replicates based on pairwise country comparison in simulation 4: (A) US vs Malawi; (B) Malawi vs Venezuela; (C): US vs Venezuela.

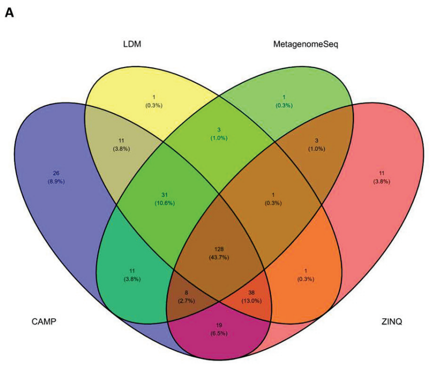

Fig. S3 Differential abundance analysis results for gut microbiome dataset: (A) number of discoveries given by 4 methods (excluding corncob and DESeq2) in the Malawi vs US comparison; and (B) US vs Venezuela comparison.

Table S2: List of 60 taxa (genus) uniquely identified by CAMP in the three pairwise comparisons (MA vs US; MA vs VE and US vs VE) with their corresponding CAMP p-values. p-values indicating statistical significance (at 5% false discovery rate) are bolded. The maximum significant p-values at 5% false discovery rate for MA vs US, MA vs VE, and US vs VE are 0.0191, 0.0110, and 0.0245, respectively. Discoveries unique to CAMP are denoted with an asterisk (*).

Genus	p-value (MA vs US)	p-value (MA vs VE)	p-value (US vs VE)
A17	$\mathbf{2.3 \times 10^{-3}}$*	0.0373	1
Actinoallomurus	$\mathbf{2.5 \times 10^{-3}}$	0.7016	**0.0110***
Alkalibacterium	0.5196	0.2849	**0.0112***
Alloiococcus	1	0.1897	$\mathbf{5.1 \times 10^{-3}}$*
Aminiphilus	$\mathbf{9.6 \times 10^{-7}}$*	$\mathbf{9.1 \times 10^{-4}}$*	1
Amycolatopsis	$\mathbf{2.2 \times 10^{-4}}$	0.4241	$\mathbf{7.1 \times 10^{-3}}$*
Aquimonas	**0.0144***	0.9142	$\mathbf{4.6 \times 10^{-3}}$
Arcanobacterium	$\mathbf{5.4 \times 10^{-10}}$	0.0146	$\mathbf{2.2 \times 10^{-3}}$*
Arsenicicoccus	$\mathbf{4.9 \times 10^{-5}}$	0.3256	**0.0129***
Asticcacaulis	1	0.2450	$\mathbf{8.9 \times 10^{-3}}$*
Azospirillum	0.0251	0.8319	**0.0184***
Bergeriella	0.0587	0.5748	$\mathbf{4.7 \times 10^{-3}}$*
Brachybacterium	$\mathbf{2.5 \times 10^{-13}}$	$\mathbf{9.8 \times 10^{-3}}$*	$\mathbf{5.1 \times 10^{-4}}$
Bradyrhizobium	$\mathbf{2.9 \times 10^{-3}}$	0.7165	$\mathbf{6.5 \times 10^{-3}}$*
Brenneria	$\mathbf{1.7 \times 10^{-3}}$*	0.0629	$\mathbf{1.1 \times 10^{-9}}$
Caldilinea	$\mathbf{2.3 \times 10^{-3}}$*	0.1196	0.0624

Continued on next page

Genus	p-value (MA vs US)	p-value (MA vs VE)	p-value (US vs VE)
Caloramator	$\mathbf{0.0151}$	$\mathbf{0.0105}$*	0.5124
CandidatusXiphinematobacter	1	0.3241	$\mathbf{0.0142}$*
Cardiobacterium	0.4580	0.2490	$\mathbf{8.1 \times 10^{-3}}$*
Caulobacter	0.1142	$\mathbf{1.1 \times 10^{-3}}$*	$\mathbf{1.3 \times 10^{-5}}$
Cedecea	$\mathbf{3.4 \times 10^{-7}}$	$\mathbf{1.0 \times 10^{-3}}$*	$\mathbf{0}$
Chelativorans	1	0.2442	$\mathbf{9.4 \times 10^{-3}}$*
Chroococcidiopsis	$\mathbf{8.2 \times 10^{-3}}$*	0.0136	$\mathbf{5.8 \times 10^{-11}}$
Cycloclasticus	1	0.1597	$\mathbf{4.0 \times 10^{-3}}$*
Dermabacter	$\mathbf{5.5 \times 10^{-7}}$	$\mathbf{0.0106}$*	0.2385
Devosia	$\mathbf{0.0160}$*	0.6159	$\mathbf{1.1 \times 10^{-3}}$
Dokdonella	1	0.2568	$\mathbf{0.0132}$*
Dyadobacter	$\mathbf{0.0120}$*	0.2277	0.0704
Endozoicimonas	1	0.2444	$\mathbf{9.6 \times 10^{-3}}$*
Escherichia	$\mathbf{3.1 \times 10^{-10}}$	$\mathbf{9.3 \times 10^{-3}}$*	$\mathbf{0}$
Ethanoligenens	0.1995	$\mathbf{6.2 \times 10^{-3}}$*	$\mathbf{1.2 \times 10^{-3}}$
Exiguobacterium	$\mathbf{1.4 \times 10^{-5}}$	0.2445	$\mathbf{0.0117}$*
Gallionella	$\mathbf{6.7 \times 10^{-3}}$*	$\mathbf{7.2 \times 10^{-5}}$	$\mathbf{0}$
HTCC	$\mathbf{5.7 \times 10^{-4}}$*	0.7174	7.6×10^{-3}
Herpetosiphon	$\mathbf{2.3 \times 10^{-3}}$*	0.0374	1
Iamia	$\mathbf{2.3 \times 10^{-3}}$*	0.0374	1
Klebsiella	$\mathbf{5.0 \times 10^{-9}}$	$\mathbf{0.0101}$*	$\mathbf{0}$
Methylophaga	$\mathbf{1.6 \times 10^{-12}}$	$\mathbf{7.3 \times 10^{-3}}$*	$\mathbf{0}$
Micrococcus	$\mathbf{0}$	$\mathbf{3.7 \times 10^{-7}}$	$\mathbf{0.0227}$*
Mitsuaria	$\mathbf{0.0169}$*	0.0178	0.7555
Nesterenkonia	$\mathbf{0.0106}$*	0.6073	$\mathbf{3.2 \times 10^{-3}}$

Continued on next page

Genus	p-value (MA vs US)	p-value (MA vs VE)	p-value (US vs VE)
Nonomuraea	$\mathbf{1.0 \times 10^{-6}}$	0.0622	**0.0206***
Ochrobactrum	0.4323	0.0600	**0.0203***
Pectinatus	**0.0188***	$\mathbf{8.7 \times 10^{-3}}$	0.2038
Pedomicrobium	1	0.2198	$\mathbf{5.7 \times 10^{-3}}$*
Peptostreptococcus	$\mathbf{3.8 \times 10^{-3}}$*	0.8274	$\mathbf{2.9 \times 10^{-3}}$
Planctomyces	$\mathbf{5.4 \times 10^{-3}}$*	0.0614	1
Polynucleobacter	0.3793	0.2619	$\mathbf{9.5 \times 10^{-3}}$*
Prevotella	**0**	$\mathbf{8.4 \times 10^{-3}}$*	**0**
Raoultella	$\mathbf{3.8 \times 10^{-14}}$	$\mathbf{1.8 \times 10^{-6}}$*	**0**
Rheinheimera	$\mathbf{7.1 \times 10^{-3}}$*	0.0169	$\mathbf{1.5 \times 10^{-11}}$
Salinibacillus	1	0.2509	**0.0122***
Salinicoccus	$\mathbf{9.7 \times 10^{-3}}$*	0.2440	0.3244
Sejongia	$\mathbf{2.9 \times 10^{-3}}$	0.7112	**0.0114***
Serratia	$\mathbf{8.0 \times 10^{-13}}$	$\mathbf{6.6 \times 10^{-4}}$*	**0**
Skermanella	0.0603	0.7316	0.$\mathbf{7.1 \times 10^{-3}}$*
Sporosarcina	0.0571	0.6001	$\mathbf{7.1 \times 10^{-3}}$*
TG5	0.7218	0.1151	**0.0185***
Terribacillus	**0.0112***	$\mathbf{9.9 \times 10^{-3}}$	$\mathbf{4.0 \times 10^{-13}}$
Xenophilus	$\mathbf{5.4 \times 10^{-3}}$*	0.5494	0.0724

S6 Sensitivity to library size

In response to a reviewer's comment that the negative log transformation depends on the library size, we explored the potential effects of differences in library sizes between groups. We modified Simulation 1 to generate the initial library size for group 1 from $\text{Unif}(10000, 20000)$, while keeping the initial

library size for group 2 as Unif(25000, 40000), thus creating non-overlapping initial library sizes. We chose simulation 1, i.e., the Dirichlet-multinomial generation process without truncation, as it can capture the variability in microbial abundance due to biological factors more effectively. As observed in Figure S4, a systematic difference in library size can result in a slightly inflated type I error (Panel A) for CAMP, although the power (Panel B) remains relatively the same.

In practical settings, substantial and systematic differences in library size between groups are uncommon, barring technical variations like batch effects. To address potential inflation in type I error due to library size differences, we propose a remedy. In cases where there is a statistically significant difference in library sizes between groups (e.g., from a t-test), one solution is to perform rarefaction before applying CAMP, thus, all the samples will have the exactly the same library size.

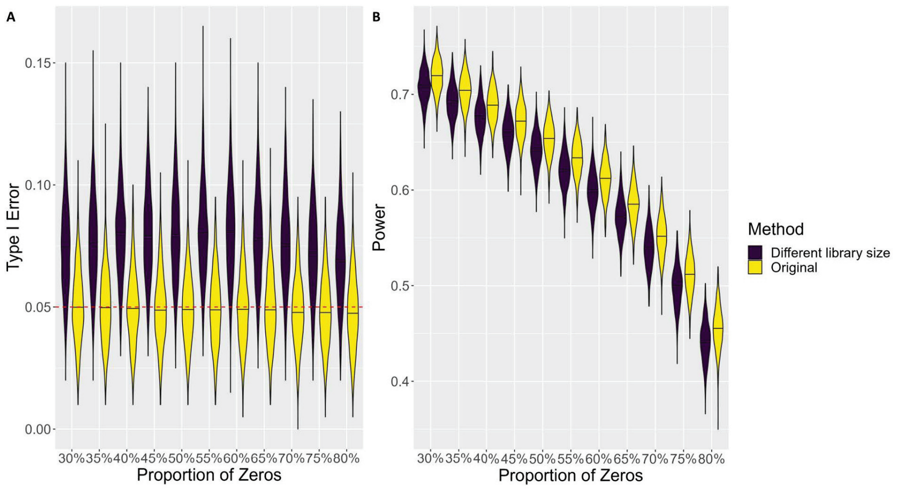

Fig. S4 Type I error and power for CAMP across 1000 replicates in a modified simulation 1: (A) Type I error; (B) Power.

S7 False discovery rate in simulation 1 and 2

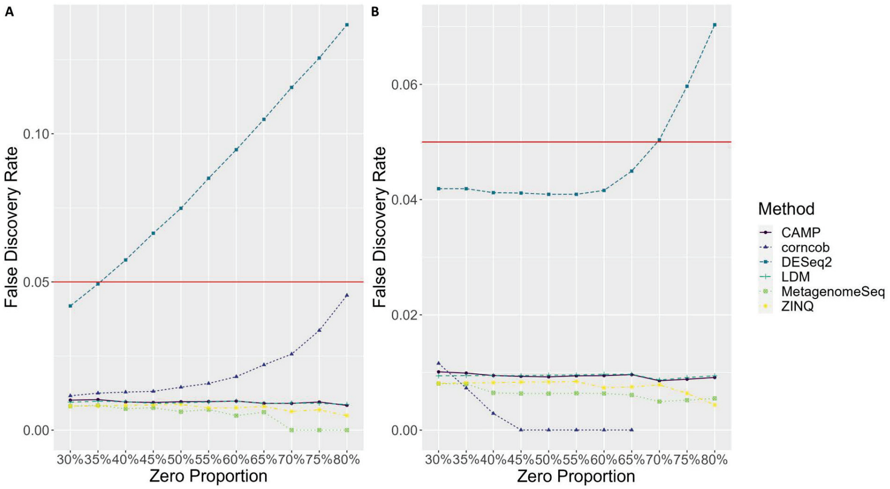

Fig. S5 Median false discovery rate and power for 6 methods compared in simulation 1 and 2 across 1000 replicates: (A) false discovery rate for simulation 1; (B) false discovery rate for simulation 2. Red horizontal line in panel A and B indicates 5% false discovery rate control.
